# Remote ischemic preconditioning STAT3-dependently ameliorates pulmonary ischemia/reperfusion injury

**DOI:** 10.1371/journal.pone.0196186

**Published:** 2018-05-16

**Authors:** Nanfu Luo, Jin Liu, Yan Chen, Huan Li, Zhaoyang Hu, Geoffrey W. Abbott

**Affiliations:** 1 Laboratory of Anesthesiology & Critical Care Medicine, Translational Neuroscience Center, West China Hospital, Sichuan University, Chengdu, Sichuan, China; 2 Bioelectricity Laboratory, Dept. of Physiology and Biophysics, School of Medicine, University of California, Irvine, California, United States of America; University of Colorado Denver, UNITED STATES

## Abstract

The lungs are highly susceptible to injury, including ischemia/reperfusion (I/R) injury. Pulmonary I/R injury can occur when correcting conditions such as primary pulmonary hypertension, and is also relatively common after lung transplantation or other cardiothoracic surgery. Methods to reduce pulmonary I/R injury are urgently needed to improve outcomes following procedures such as lung transplantation. Remote liver ischemic preconditioning (RLIPC) is an effective cardioprotective measure, reducing damage caused by subsequent cardiac I/R injury, but little is known about its potential role in pulmonary protection. Here, we analyzed the efficacy and mechanistic basis of RLIPC in a rat model of pulmonary I/R injury. RLIPC reduced lung I/R injury, lessening structural damage, inflammatory cytokine production and apoptosis. In addition, RLIPC preserved pulmonary function compared to controls following lung I/R injury. RLIPC stimulated phosphorylation of pulmonary STAT3, a component of the SAFE signaling pathway, but not phosphorylation of RISK pathway signaling proteins. Accordingly, STAT3 inhibition using AG490 eliminated the pulmonary protection afforded by RLIPC. Our data demonstrate for the first time that RLIPC protects against pulmonary I/R injury, via a signaling pathway requiring STAT3 phosphorylation.

## Introduction

Ischemia/reperfusion (I/R) injury occurs when oxygen supply to a tissue is interrupted and then reestablished. As damaging as the ischemia is, the reintroduction of oxygen—although essential—exacerbates injury by a variety of mechanisms including ionic overload and introduction of inflammatory mediators and reactive oxygen species. I/R injury can occur when therapeutically establishing oxygen supply after a pathologic ischemic episode, e.g., with thrombolytic therapy or primary percutaneous coronary intervention following a myocardial infarction [1], or with thromboendarterectomy following pulmonary artery embolism [2]. Alternatively, I/R injury can result when both the ischemia and the reperfusion result from a therapeutic intervention, such as in lung transplantation.

The lungs are unique in that they acquire oxygen both directly from the atmosphere via alveolar gas exchange, as well as via the blood in the form of a dual circulatory system comprising the bronchial and pulmonary arteries [3]. Pulmonary ischemia is therefore also somewhat different to that in other organs. Ventilated pulmonary ischemia describes a situation in which alveolar oxygen supply remains but blood supply is interrupted, such as occurs in pulmonary artery embolisms or primary pulmonary hypertension. Anoxic pulmonary ischemia refers to cessation of both alveolar and blood oxygen supply, as occurs during lung transplantation, or in the pulmonary collapse employed during lung lobectomy [4, 5].

Pulmonary I/R injury is a major factor in lowering success rates of lung transplantation, because it is the major cause of primary graft dysfunction (PGD)[6], affecting around 15% of patients undergoing lung transplants[3]. PGD, in turn, increases the risk of late graft rejection, the main cause of late (>12 months post-surgery) lung transplant rejection [7]. As lung transplantation surgery is becoming more common, and there are currently no specific therapeutic interventions to prevent pulmonary I/R injury, it is accepted that novel therapeutic approaches are needed to diminish the effects of pulmonary I/R injury and thus reduce the incidence of PGD [8].

Remote ischemic preconditioning refers to an intervention in which ischemia is induced in one organ or region of the body to induce a protective effect in another organ undergoing I/R injury. Several reports indicated that limb ischemic conditioning protects lungs [9, 10]. Interestingly, we and other found that brief ischemic preconditioning of visceral organs such as the liver, the largest metabolic organ in the body, exerts strong cardioprotection [11–16]. The question still remains whether this brief liver ischemia-induced preconditioning can raise the tolerance to reperfusion-induced lung injury, and if so, can we identify an underlying molecular mechanism that could be potentially be leveraged therapeutically in the future. Here, we describe application of remote liver ischemic preconditioning (RLIPC) to a rat pulmonary I/R injury model, and report an underlying molecular mechanism for its protective effect.

## Materials and methods

### Animals

All experimental protocols and procedures in the current study were used according to the recommendations in the Guide for the Care and Use of Laboratory Animals of the National Institutes of Health (8th edition, 2011). The Institutional Animal Care and Use Committee of Sichuan University (Sichuan, China) approved the study (Permit Number: 2015035A). Male Sprague Dawley rats of Specific Pathogen Free (SPF) grade and bred for research purposes were purchased from Dashuo Laboratory Animal Reproduction Center (Chengdu, China). Rats (250–300 g) of 7–8 weeks of age were used, and were housed at 20–25°C with a humidity of 60 ± 5% under a 12-h light–dark cycle.

### Experimental protocol

**Fig 1:**
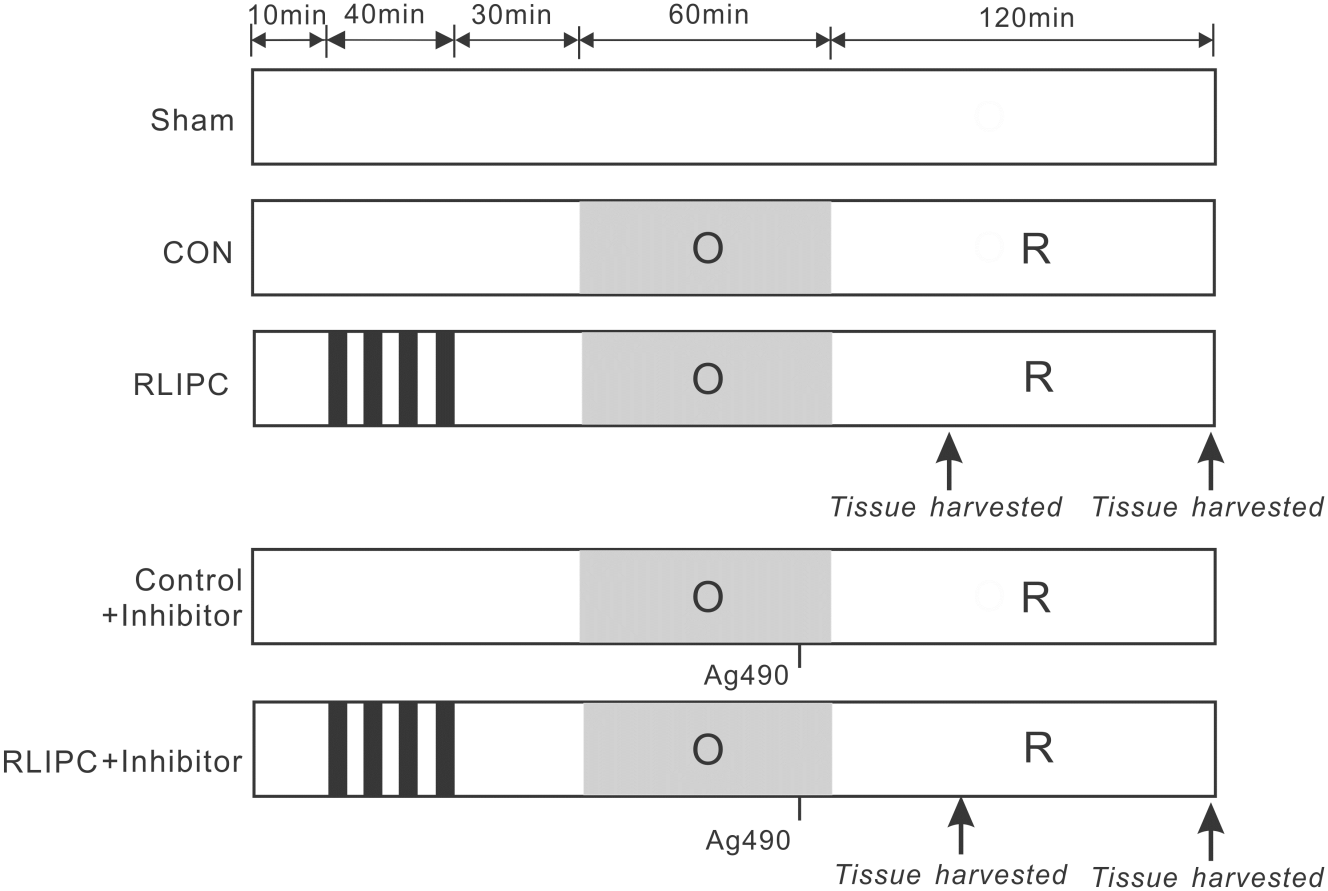
Experimental protocols. All lungs were subjected to a 60-min ligation of the left pulmonary hilus (O), followed by 2 h of reperfusion (R), except for the sham-operated rats. For remote liver ischemic preconditioning (RLIPC), four cycles of 5 min of liver ischemia with 5min intermittent reperfusions were conducted before pulmonary ischemia. Thirty minutes of acute memory phase were allowed before being followed by a 60-min left pulmonary hilus ischemia. The STAT3 inhibitor Ag490 was applied 5 min before reperfusion. Arrows indicate the time points at which tissue samples were harvested.

Fig 1 depicts the experimental procedure. Rats were assigned to a sham-operated group (”Sham”, in which the hepatic arterial and venous trunk were exposed without intervention, and lung was exposed without ligation), control group (CON, no further hepatic intervention) or remote liver ischemic preconditioning group (RLIPC) group. For RLIPC, after laparotomy, the portal vein, hepatic arterial and venous trunk were identified and clamped with microvascular clip. RLIPC was exerted by four cycles of 5 min each of liver ischemia with 5 min intermittent reperfusions. AG490, a STAT3 inhibitor (5 mg/kg, Sigma, St. Louis, MO, USA) was intravenously administrated via the femoral vein 5 min prior to pulmonary reperfusion.

### Surgical procedures

10 minutes were allowed for stabilization after surgical preparation. Details of the surgical implantation of instruments have been described previously [13, 17]. Briefly, sodium pentobarbital (50 mg/kg, intraperitoneal injection) was used for anesthesia. The loss of the corneal reflex, the lack of response to toe-pinching and heart rate were indicators for anesthesia adequacy. Rats were intubated and ventilated with a rodent ventilator (Taimeng, Chengdu, China). Tidal volume was 8–10 ml, frequency was 60–70 times per minute, and expiration:inspiration was 5:4. During the clamping time of the lung, tidal volume was 6 ml, frequency was 70–80 times per minute, with the same expiration:inspiration (5:4). After performing a left anterolateral thoracotomy, the chest was opened and the left hilum including the pulmonary artery, vein, and main stem bronchus were clamped with a microvascular clip. Lungs were occluded for a period of 60min, followed by reperfusion for 2h. The left pulmonary hilum was not occluded in sham-operated rats. At the end of the reperfusion, rats were euthanized with an overdose of sodium pentobarbital (200 mg/kg, i.p.).

### Blood gas measurement

The portion of the right carotid artery next to the trachea was isolated. A 24-G heparin-filled catheter (Spacelabs Medical, Inc., Redmond, WA, USA) was inserted into the right carotid artery for arterial blood collection. Arterial blood (0.3ml) was collected at each time point, for blood gas measurement using a blood gas analyzer (ABL800 FLEX, Radiometer Medical A/S, Copenhagen, Denmark).

### Serum preparation

At the end of the experiment, blood samples were immediately taken from the left ventricles and then centrifuged at 4,000 g for 10 min at 4°C. The supernatant was then taken and frozen at -20°C for further assessment.

### BAL fluid collection and cell counts

At the end of 2h reperfusion, lungs were lavaged three times with 1.5 ml of ice-cold, sterile PBS. Bronchoalveolar lavage (BAL) fluids were collected via the intratracheal cannula and were then centrifuged at 1,500 *g* for 10 min at 4°C. Supernatant fluids were stored at -80°C for subsequent ELISA analysis. Cell pellets were resuspended and recovered by centrifugation and counted with the aid of a hemocytometer in a double-blind manner.

### Determination of cytokine content

Tumor necrosis factor-α (TNF-α) and interleukin-6 (IL-6) in BAL were quantified using an ELISA kit (Develop, Wuxi, Jiangsu, China) according to the manufacturer’s instructions.

### Myeloperoxidase (MPO) activity assay

MPO activity in the left lung tissue was measured using assay kits (Jiancheng Bioengineering Institute, Nanjing, China) according to the manufacturer’s instructions.

### Wet lung/Dry lung weight ratio

After the experiment, the left lower lobe of the lung was removed and the wet weight was determined immediately. It was then dried in an oven at 60°C for 72 hours and the weight was re-measured. The wet and dry weights were then used to calculate the percent water content: (wet weight-dry weight) / wet weight x 100.

### Lung tissue collection

The left lung of each rat was fixed with 10% formaldehyde overnight and then embedded in paraffin wax. Lung slices were cut into 5μm sections and were mounted on glass slides before been stained with hematoxylin and eosin (H&E) or terminal deoxynucleotidyl transferase mediated dUTP nick-end labeling (TUNEL) for the assessment of lung edema or apoptosis.

### Histological evaluation

The histological changes were evaluated based on a lung injury scoring system[18]. Briefly, the degree of pulmonary damage was graded as: 0: no neutrophils in the alveolar/ interstitial space, no hyaline membranes, no proteinaceous debris filling the airspaces; <2x alveolar septal thickening; 1: 1–5 neutrophils in the alveolar/interstitial space, 1 hyaline membranes, 1 proteinaceous debris filling the airspaces, 2x–4x alveolar septal thickening; 2:>5 neutrophils in the alveolar/interstitial space, >1 hyaline membranes, >1 proteinaceous debris filling the airspaces, >4x alveolar septal thickening. The final histological score = [(20 x A) + (14 x B) + (7 x C) + (7 x D) + (2 x E)]/(number of fields x 100).

### TUNEL assay

Terminal deoxynucleotidyl transferasemediated dUTP nick-end labeling (TUNEL) assay was adopted to evaluate cell apoptosis according to the manufacturer’s instructions (Roche Applied Science, Indianapolis, IN). Ten different fields from each slice were chosen at random and blindly analyzed. TUNEL-positive apoptotic nuclei were stained fluorescent green. The apoptotic index was judged as a percent of the number of TUNEL-positive nuclei to the total nuclei population. A fluorescence microscope (Zeiss, Oberkochen, Germany) was used for image evaluation.

### Western blot analysis

Left lungs were surgically dissected and immediately frozen 30min post-I/R. Frozen left lung tissue samples were homogenized with RIPA buffer contained 50 mM Tris-HCl (pH7.4), 150 mM NaCl, 1% NP-40, 1 mM EDTA, 0.25% sodium deoxycholate, mixed with phosphatase inhibitor cocktail (Sigma-Aldrich, St. Louis, MO, USA), and a protease inhibitor cocktail (Sigma-Aldrich, St. Louis, MO, USA). The homogenate was centrifuged at 10,000 *g* for 10 min at 4°C. Protein concentration was determined by BCA (Pierce, Rockford, IL, USA). An equal amount of protein (15 μg) was separated by 12% SDS-PAGE and transferred onto nitrocellulose membranes (VWR, Batavia, IL, USA). Primary rabbit antibodies included those raised against: phosphorylated extracellular signal-regulated kinase1/2 (ERK1/2) (Thr202/Tyr204) (p-ERK), total ERK1/2, phosphorylated Akt (Ser473) (p-AKT), total Akt, phosphorylated glycogen synthase kinase-3β (Ser9) (p-GSK-3β), total -GSK-3β, phosphorylated STAT3(Tyr705) (p-STAT3), and total STAT3, (all, 1:1000, Cell Signaling, Danvers, MA, USA). Horseradish peroxidase (HRP)-conjugated goat anti-rabbit IgG was used as secondary antibody (Bio-Rad, Hercules, CA, USA). With a chemiluminescence system (Millipore, Billerica, MA, USA), signals were visualized using an AmershamImager 600 system (GE Healthcare, Little Chalfont, UK) and band densities were determined by ImageJ Data Acquisition Software (National Institutes of Health, Bethesda, MD, USA). Band density ratios were calculated.

### Statistical analysis

All data were expressed as mean ± SEM. The unpaired two-tailed student’s t-tests were used for the comparison of data between two groups. For multiple comparisons over three groups, one-way ANOVA was used followed by Newman-Keuls test or Dunnett’s T3 test depending on the equality of homogeneity of variance. P<0.05 was considered to be statistically significant.

## Results

### RLIPC reduces lung damage associated with pulmonary I/R injury

**Fig 2:**
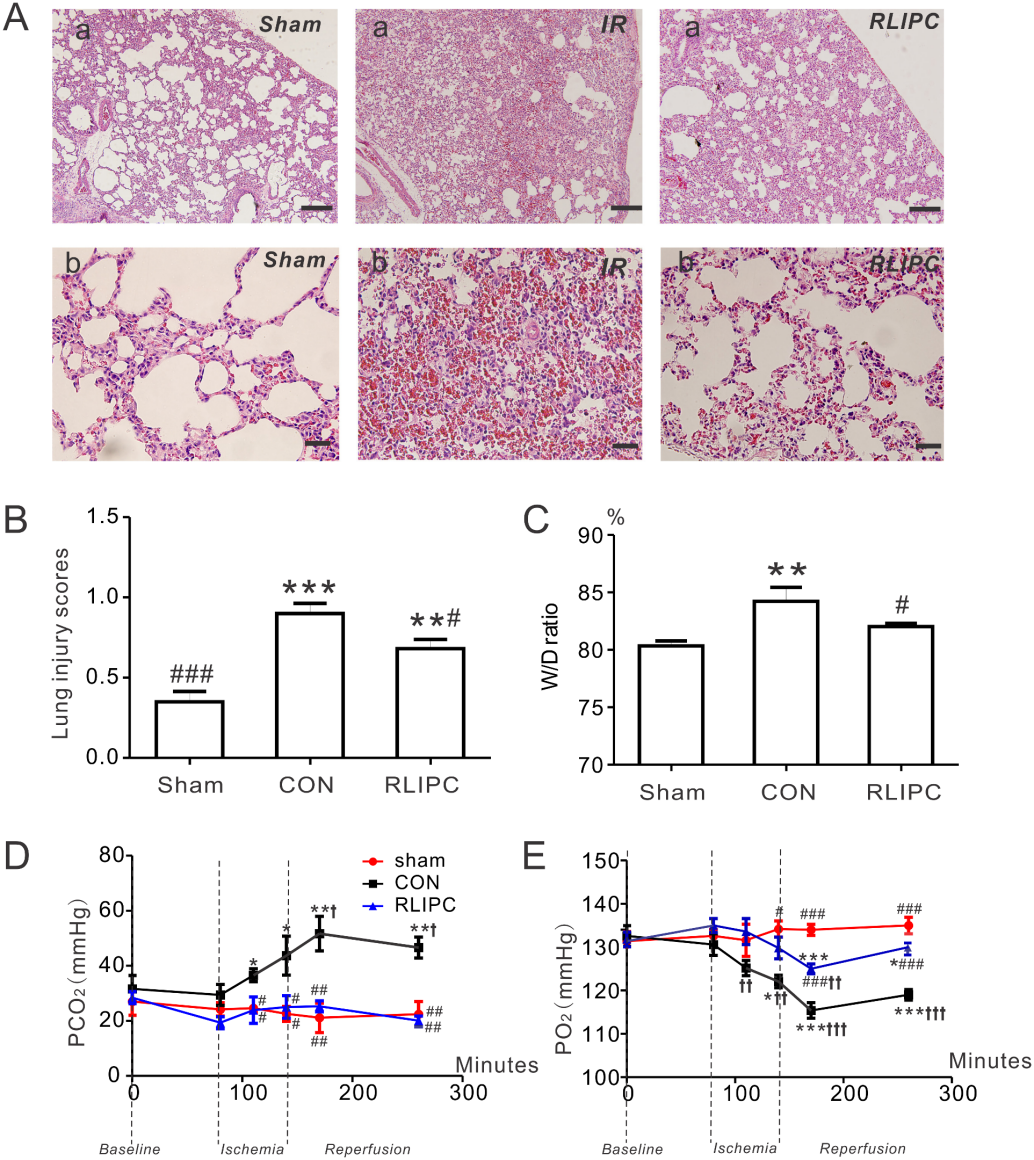
RLIPC ameliorates pulmonary injury after experimentally imposed pulmonary IR. ***A***. Representative images of hematoxylin and eosin-stained left lung sections. Lung histopathology changes were examined under a light microscope; *n* = 4–5 rats per group. Panel (a) scale bars: 100 μm; panel (b) scale bars, 20 μm. ***B***. Morphological evaluation of lung sections of sham-operated, CON and RLIPC rats. Sham animals did not undergo liver stimulus; CON, control, RLIPC, remote liver ischemic preconditioning; *n* = 4–5 rats per group. **P<0.01, ***P<0.001, compared with sham-operated lungs, ^#^P<0.05 compared with CON (by One-way ANOVA). ***C***. Water content (%) of left lung sections from sham-operated, CON and RLIPC rats post-pulmonary I/R injury; *n* = 6–7. All data were expressed as mean ± SEM. **P<0.05 compared with sham-operated lungs, ^#^P<0.05 compared with CON (by One-way ANOVA). ***D***. Changes in PCO_2_ values from sham-operated, CON and RLIPC rats; *n* = 5–7, *P<0.05, **P<0.01, compared with sham-operated lungs, ^#^P<0.05,^##^P<0.01, compared with CON, ^†^P<0.05 compared with baseline. ***E***. Changes in PO_2_ values from sham-operated, CON and RLIPC rats; *n* = 5–7, *P<0.05, ***P<0.001, compared with sham-operated lungs, ^#^P<0.05,^###^P<0.001, compared with CON,^††^P<0.01, ^†††^P<0.001 compared with baseline.

Hematoxylin and eosin staining (Fig 2A) on sham-operated lung sections showed an approximately normal histological structure with little hemorrhage, or neutrophil infiltration. Additional raw data images for these and all other micrographs and western blots are provided in S1 Fig. In contrast, I/R injury caused significantly alveoli structure damage, such as inter-alveolar septum thickening, pulmonary edema, evident inflammatory cell infiltration, and alveolar bleeding (P<0.01 or P<0.001 vs sham, Fig 2B). RLIPC was effective at reducing lung I/R injury-associated alveolar damage, indicated by lower lung injury scores in the RLIPC group compared with those in the non-preconditioned (CON) group (P<0.05, Fig 2B). Wet-to-dry lung weight ratios were quantified as an index of lung water accumulation in rats subjected to 1 h of left lung ischemia followed by 2h of reperfusion. Demonstrative of lung pathology, the wet-to-dry ratio in the control group (6.5 ± 0.5) was significantly higher than that in the sham-operated (5.1 ± 0.1) or RLIPC (5.6 ± 0.1) groups (P<0.05); in contrast, no difference was found between the wet-to-dry lung ratios of the sham and RLIPC groups after pulmonary IR injury (P>0.05), indicating RLIPC was effective at preventing lung I/R injury-associated edema (Fig 2C).

Fig 2D and 2E illustrate the time course of changes of blood pCO_2_ and pO_2_ during I/R injury. RLIPC was highly effective at preserving lung function, completely preventing the marked (>twofold) I/R injury-associated increase in pCO_2_ (compared to sham-operated) observed in rats without RLIPC (Fig 2D). Similarly, RLIPC greatly attenuated the I/R injury-associated reduction in pO_2_ observed in rats without RLIPC (Fig 2E).

### Inflammatory cytokine production

**Fig 3:**
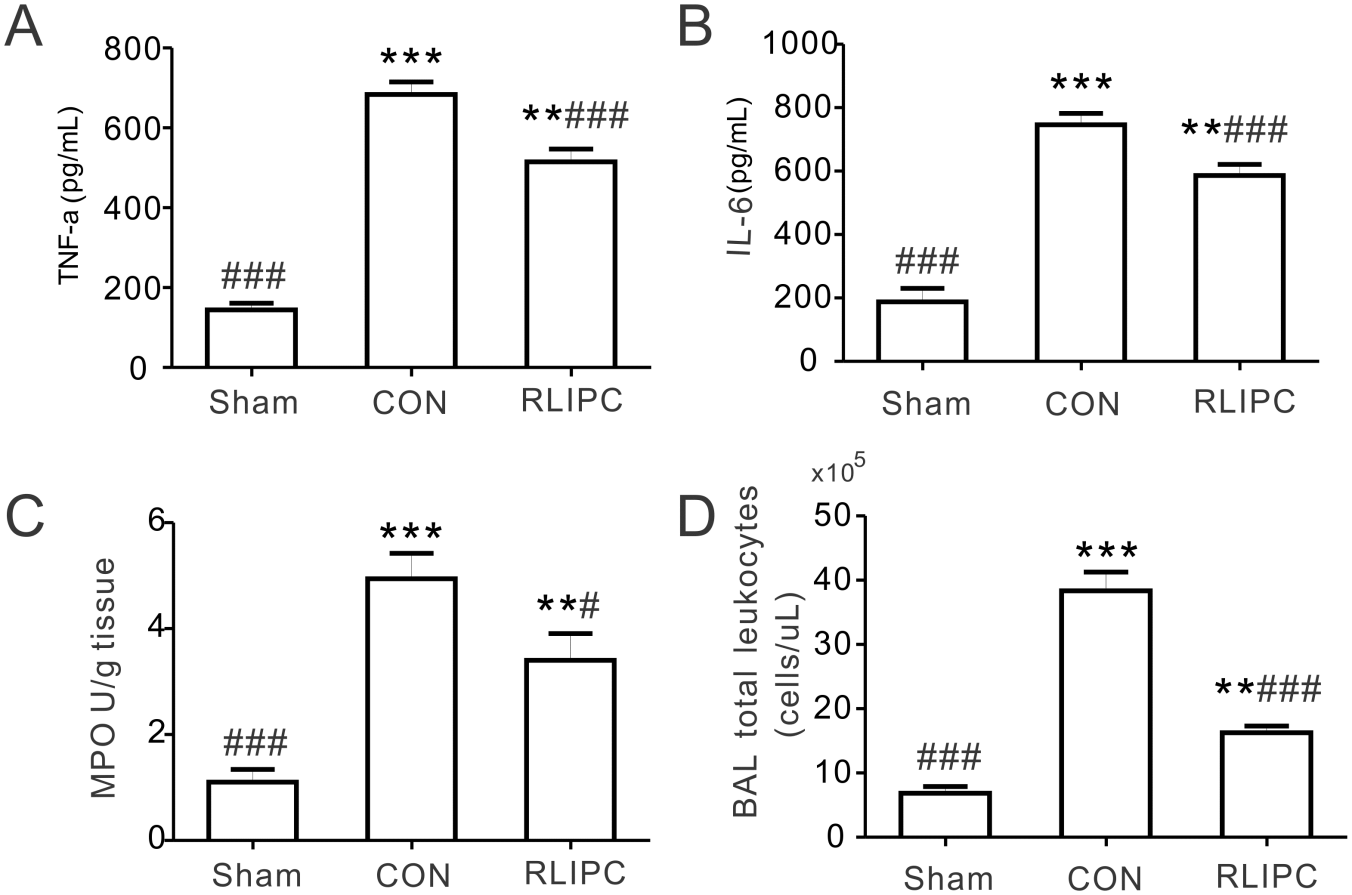
RLIPC decreases the production of inflammatory mediators after pulmonary I/R injury. ***A***. TNF-α content in BAL fluids at the end of 2 h of reperfusion from sham-operated, CON and RLIPC rats; *n* = 6 rats per group. Sham, sham-operated group; CON, control; RLIPC, remote liver ischemic preconditioning. **P<0.01, ***P<0.001 compared with sham-operated lungs, ^###^P<0.001, compared with CON (by One-way ANOVA). ***B***. IL-6 content in BAL fluids at the end of 2h of reperfusion from sham-operated, CON and RLIPC rats; *n* = 6 rats per group. **P<0.01, ***P<0.001 compared with sham-operated lungs, ^###^P<0.001, compared with CON (by One-way ANOVA). ***C***. Lung tissue harvested at the end of 2h reperfusion from sham-operated, CON and RLIPC rats were homogenized and assessed for myeloperoxidase (MPO) activity; *n* = 6 rats per group. **P<0.01, ***P<0.001 compared with sham-operated lungs, ^#^P<0.05, ^###^P<0.001, compared with CON (by One-way ANOVA). ***D***. Total leukocytes were quantitated in BAL fluids at the end of 2h of reperfusion from sham-operated, CON and RLIPC rats; *n* = 5 rats per group. **P<0.01, ***P<0.001, compared with sham-operated lungs, ^###^P<0.001 compared with CON (by One-way ANOVA).

TNF-α and IL-6, the major inflammatory cytokines, are released in the early stage of I/R injury in the lung and further stimulate neutrophils to release inflammatory mediators [8, 19, 20]. Statistically significant differences in TNF-α and IL-6 levels were detected among the experimental groups (TNF-α: P = 0.0001; IL-6: P = 0.0001). Pulmonary reperfusion caused an elevation of TNF-α and IL-6 levels in both the CON and the RLIPC groups (P<0.01 or P<0.001 vs. sham-operated rats), but the increase was less in RLIPC versus CON (P<0.001) (Fig 3A and 3B). As an indicator of lung inflammation, MPO activity was also quantified to assess the degree of lung neutrophil accumulation. Compared with the sham-operated group (1.1 ± 0.2 U/g), reperfusion injury caused a statistically significant elevation of MPO activity in both the CON and RLIPC groups (P<0.01 or P<0.001), but liver preconditioning lessened the increase in I/R-induced MPO activity (RLIPC, 3.4 ± 0.5 U/g; P<0.05 vs. CON, 4.9 ± 0.5 U/g; Fig 3C). Consistent with this, RLIPC dramatically reduced the total number of leukocytes found in the BAL fluid, compared with that in control lungs (Fig 3D, P<0.001).

### Liver ischemic preconditioning reduced lung apoptosis post I/R

**Fig 4:**
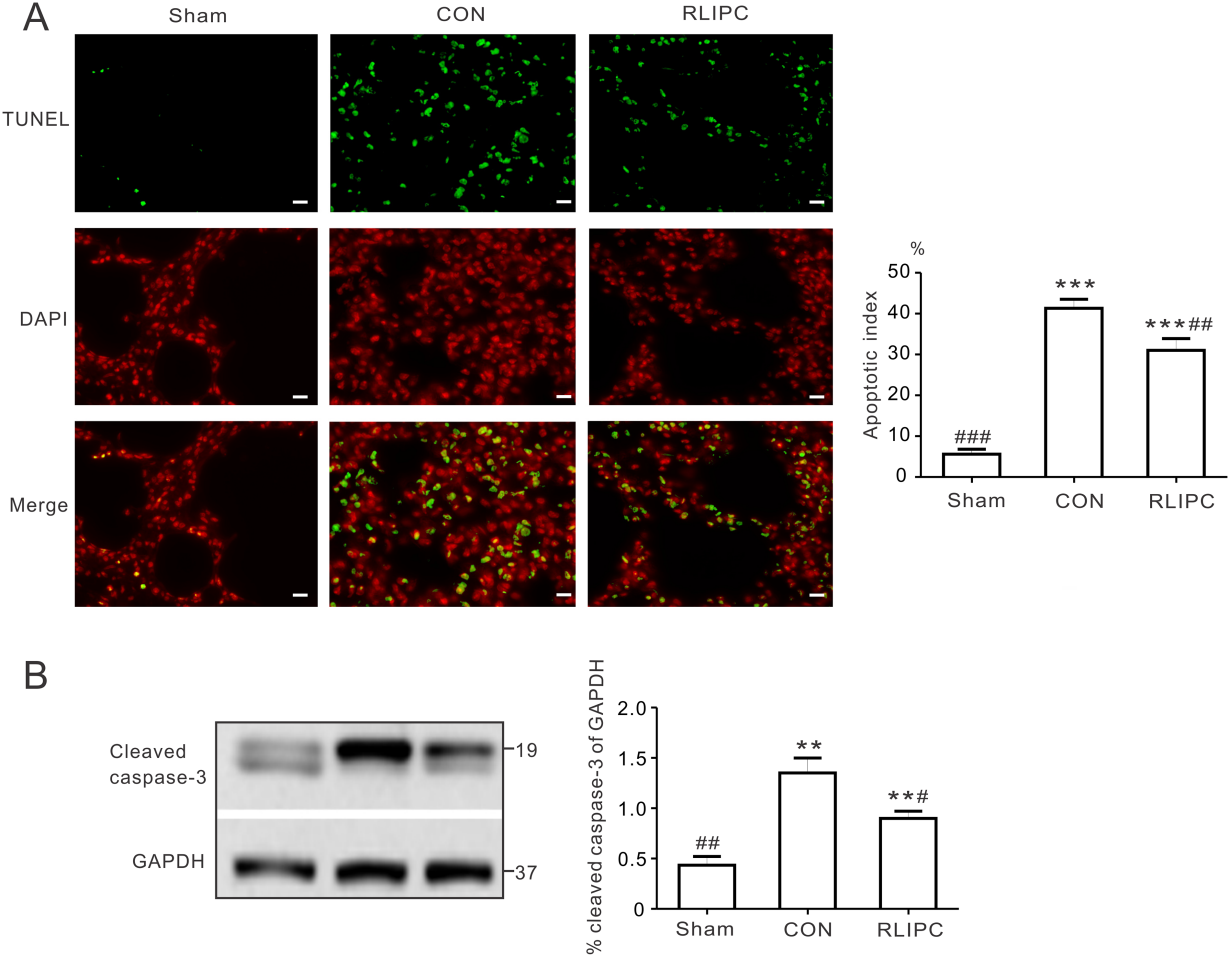
RLIPC reduces pulmonary apoptosis post I/R. ***A***. Terminal transferase dUTP nick end labeling (TUNEL) technique was applied for the analysis of pulmonary apoptosis. ***Left***, TUNEL-positive nuclei in the left lungs obtained from sham-operated (sham), liver conditioned (RLIPC) and control (CON) rats after pulmonary reperfusion injury were stained green (TUNEL) and nuclei were stained red (DAPI). Scale bars, 20μm. ***Right***, Bar graph showing the TUNEL-positive nuclei expressed as a percentage of total nuclei in the left lung sections; *n* = 4–6. All data were expressed as mean ± SEM. ***P<0.001 compared with sham-operated lungs, ^##^P<0.01, ^###^P<0.001, compared with CON (by One-way ANOVA). ***B***. Representative Western blots (***left***) and quantification (***right***) of cleaved caspase-3 protein band density (normalized to GAPDH) in sham, CON, and liver-conditioned rat left lungs are shown; *n* = 5. **P<0.*05*, compared with sham-operated rats; ^*#*^*P<0*.*05*, ^*##*^*P<0*.*01*, compared with CON (by One-way ANOVA).

TUNEL-positive nuclei were prevalent in the left lungs post I/R injury (P<0.001 vs. sham), indicating extensive induction of apoptosis. However, RLIPC reduced the number of TUNEL-positive stained apoptotic nuclei (31.0 ± 2.9%) compared with non-RLIPC treated CON (41.3 ± 2.2%, P<0.01, Fig 4A). Caspase-3 plays a key role in mediating apoptotic cell death, requiring cleavage of caspase-3 by an initiator caspase [21]. Here, I/R caused threefold elevation of cleaved caspase-3 expression compared with the sham-operated group (P<0.01). In agreement with the changes in apoptosis, RLIPC attenuated the I/R-induced increase in cleaved caspase-3 (P<0.05. vs. CON) (Fig 4B). Together, these data indicate that pulmonary I/R-induced lung apoptosis is ameliorated by RLIPC.

### Effects of RLIPC on pulmonary RISK and SAFE pathway induction post-IR

**Fig 5:**
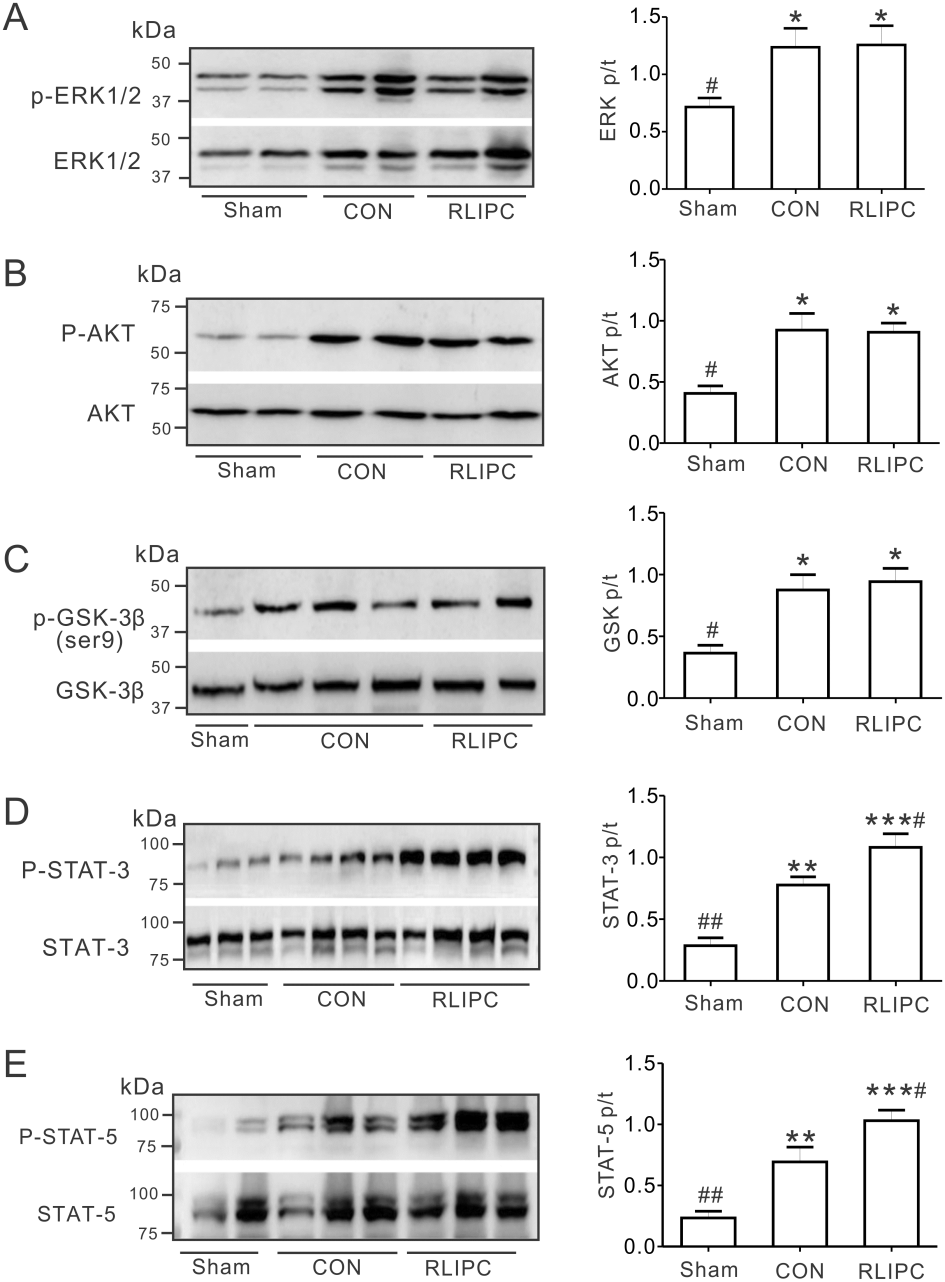
RLIPC affects pulmonary STAT3 pathway induction. ***A***. Western blots (*left*) and quantification (*right*) of ERK1/2 (p) and ERK1/2 (t) protein band density in sham, CON, and liver-conditioned rat left lungs are shown; *n* = 5–7. *P<0.*05*, compared with sham-operated rats; ^*#*^*P<0*.*05*, compared with CON (by One-way ANOVA). ***B***. *Left*, representative western blots of phospho- (p) AKT and total (t) AKT isolated from sham, CON, and liver-conditioned rat left lungs; one rat per lane. *Right*, mean ratio of pAKT/tAKT band density from blots as in left; *n* = 7. *P<0.*05*, compared with sham-operated rats; ^*#*^*P<0*.*05*, compared with CON (by One-way ANOVA). ***C***. *Left*, representative western blots of phospho- (p) GSK3β and total (t) GSK3β isolated from sham, CON, and liver-conditioned rat left lungs. *Right*, mean ratio of pGSK3β/tGSK3β band density from blots as in left; *n* = 6. *P<0.*05*, compared with sham-operated rats; ^*#*^*P<0*.*05*, compared with CON (by One-way ANOVA). ***D***. *Left*, representative western blots of phospho- (p) STAT3 and total (t) STAT3 isolated from sham, CON, and liver-preconditioned rat left lungs. *Right*, mean ratio of pSTAT3/tSTAT3 band density from blots as in left; *n* = 6. **P<0.*01*, ***P<0.*001* compared with sham-operated rats; ^*#*^*P<0*.*05*, ^*##*^*P<0*.*01* compared with CON (by One-way ANOVA). ***E***. *Left*, representative western blots of phospho- (p) STAT5 and total (t) STAT5 isolated from sham, CON, and liver-preconditioned rat left lungs. *Right*, mean ratio of pSTAT5/tSTAT5 band density from blots as in left; *n* = 5–6. **P<0.*01*, ***P<0.*001* compared with sham-operated rats; ^*#*^*P<0*.*05*, ^*##*^*P<0*.*01* compared with CON (by One-way ANOVA).

We previously showed that RLIPC exerted its cardioprotective effect by activating the reperfusion injury salvage kinase (RISK) pathway [12, 13, 16]. To further understand the RLIPC-induced lung protective effect, we investigated whether or not RLIPC induced the RISK pathway post-pulmonary I/R, specifically by quantifying ERK1/2, AKT, and GSK-3β protein phosphorylation (Fig 5A–5C). We normalized the levels of each phosphorylated form to their corresponding total protein levels. No differences were detected in total protein levels of ERK1/2, AKT, and GSK-3β in any of the experimental groups (P>0.05). Surprisingly, after pulmonary I/R, RISK pathway induction was similar in control and RLIPC-treated rats (P>0.05), i.e., compared to control hearts, RLIPC equally increased phosphorylation of pulmonary ERK1/2, AKT and GSK-3β(Ser9) (all, P<0.05 vs. sham-operated lungs), suggesting that an alternative signaling pathway might be responsible for the RLIPC-induced lung protection that we observed. Activation of the survivor activating factor enhancement (SAFE) pathway, including the signal transducer and activator of transcription 3 (STAT3) signaling protein, can also reportedly be involved in ischemic preconditioning [22]. We therefore assessed its possible involvement, and found that the ratio of phosphorylated to total pulmonary STAT3 protein was higher in control rats (0.8 ± 0.1) than in sham-operated rats (0.3 ± 0.1, P<0.01), and was further enhanced (to 1.1 ± 0.1, P<0.05 vs. CON) in the RLIPC lungs (Fig 5D). We observed a similar pattern for STAT5 (Fig 5E), which has been implicated in cardioprotection by RIPC [23, 24].

### Inhibition of STAT3 abolishes the protective action of liver preconditioning

**Fig 6:**
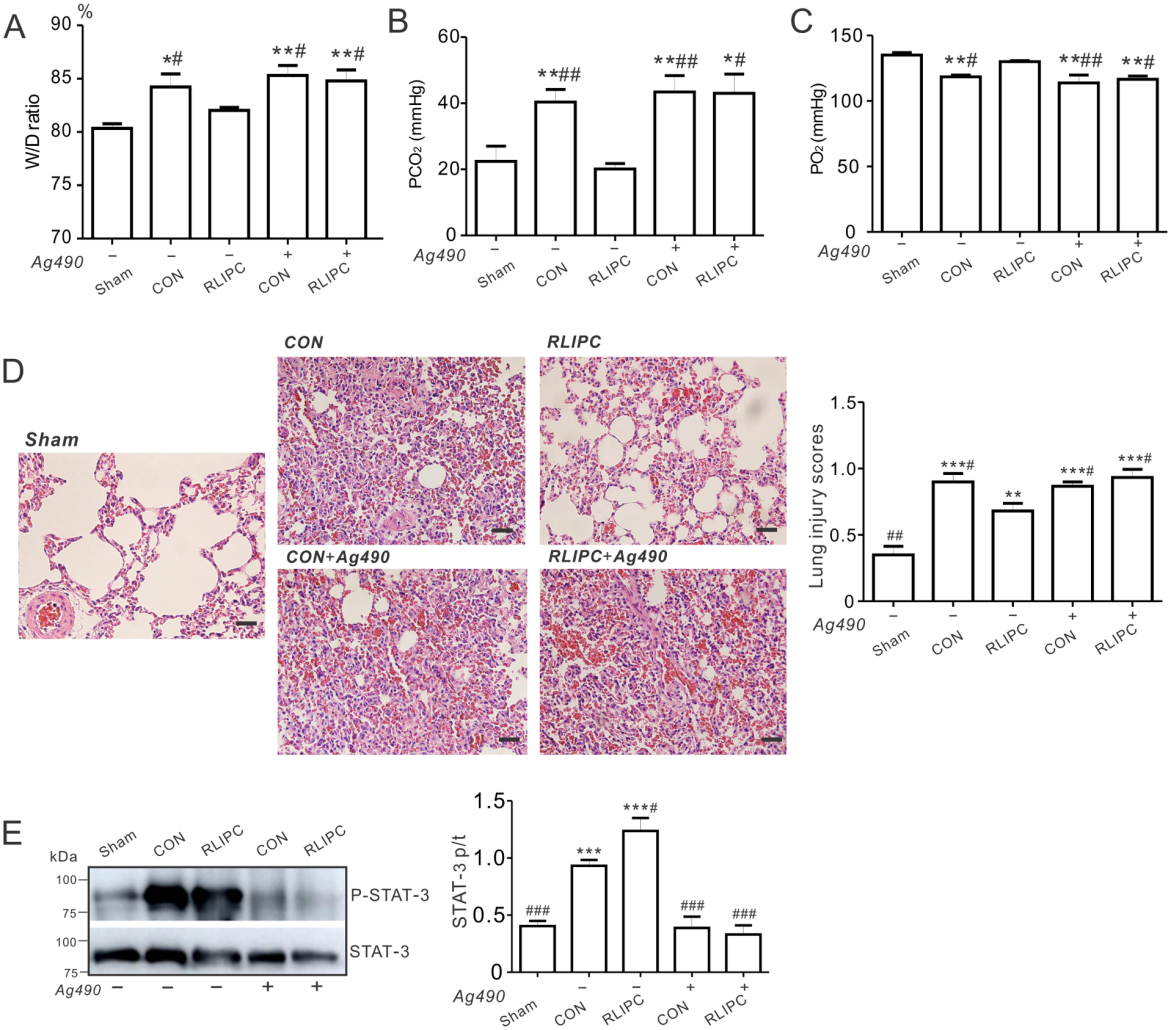
Pharmacological inhibition of STAT3 abolishes the RLIPC-induced protection against pulmonary I/R injury. ***A***. Water content (%) of left lung sections from sham-operated, CON and RLIPC rats with (+) or without (-) Ag490 post-pulmonary I/R injury; *n* = 6–7. Values for sham-operated, CON and RLIPC rats are repeated from Fig 2C for comparison. Sham, sham-operated group; CON, control; RLIPC, remote liver ischemic preconditioning. W/D ratio was expressed as wet weight-dry weight) / wet weight x 100. All data were expressed as mean ± SEM. *P<0.05, **P<0.01, compared with sham-operated lungs, ^#^P<0.05 compared with RLIPC lungs without Ag490 (by One-way ANOVA). ***B***. Changes in PCO_2_ values from left lungs of rats with (+) or without (-) Ag490 after 180 min of pulmonary I/R injury; *n* = 6–7. Values for sham-operated, CON and RLIPC rats are repeated from Fig 1D for comparison. All data were expressed as mean ± SEM. *P<0.05, **P<0.01, compared with sham-operated lungs, ^#^P<0.05, ^##^P<0.01, compared with RLIPC lungs without Ag490 (by One-way ANOVA). ***C***. Changes in PO_2_ values from left lungs of rats with (+) or without (-) Ag490 after 180 min of pulmonary I/R injury; *n* = 6–7. Values for sham-operated, CON and RLIPC rats are repeated from Fig 1E for comparison. All data were expressed as mean ± SEM. **P<0.01, compared with sham-operated lungs, ^#^P<0.05, ^##^P<0.01, compared with RLIPC lungs without Ag490 (by One-way ANOVA). ***D*. *Left***, representative histological H&E-stained micrographs of lung sections from rat left lungs with or without Ag490 post-IR. Representative of *n* = 4–5 rats per group. Scale bars, 20 μm. ***Right***, morphological evaluation of pulmonary damage after reperfusion in left lungs (*n* = 4–5 rats per group). The type and severity of pulmonary damage were graded as shown in the ‘Methods’ section. Values for CON and RLIPC rats are repeated from Fig 2*B* for comparison. All data were expressed as mean ± SEM. **P<0.01, ***P<0.001, compared with sham-operated lungs, ^#^P<0.05 compared with RLIPC lungs without AG490 (by One-way ANOVA). ***E*. *Left***, representative Western blots of phospho- (p) STAT3 and total (t) STAT3 isolated from left lungs of rats with (+) or without (-) Ag490. ***Right***, mean ratio of pSTAT3/tSTAT3 band density from blots as in left; *n* = 5–6 each group. ***P<0.*001* compared with sham-operated lungs; ^*#*^*P<0*.*05*, ^*###*^*P<0*.*001* compared with CON (by One-way ANOVA).

Inhibition of STAT3 (by AG490) inhibits STAT3 phosphorylation and abolishes ischemic preconditioning-induced cardioprotection [25]. To further explore the role of STAT3 in RLIPC-mediated pulmonary protection, we next asked if administration of AG490 prior to reperfusion injury during the lung ischemic period could affect lung function and injury in our model. Non-AG490 pretreated RLIPC rats were less susceptible to lung injury after reperfusion compared to control rats. Strikingly, AG490 pretreatment abolished the pulmonary protective effects we had previously observed for RLIPC rats, such that inhibitor-treated RLIPC and control rats exhibited equal elevation of lung W/D ratios after reperfusion, to levels similar to that of control lungs without inhibitor treatment (P<0.05 or P<0.01 vs. sham, Fig 6A). Additionally, AG490 eliminated the decrease in pCO_2_ that was achieved by RLIPC without AG490 pretreatment (P<0.05 vs RLIPC alone, Fig 6B). Consistent with this, AG490-pretreated RLIPC rats exhibited decreased post-I/R pO_2_ compared to rats exposed to RLIPC alone (P<0.05, Fig 6C). Furthermore, inhibition of STAT3 using AG490 in the RLIPC group produced excessive pulmonary structural damage compared with what we observed in RLIPC alone, inducing damage similar to that in control lungs (P<0.05 vs RLIPC alone, Fig 6D). Confirming the expected molecular action of AG490, RLIPC-induced phosphorylation of STAT3 was completely blocked by applying AG490 (3.7-folder lower than RLIPC alone), diminishing it to a level similar to that of lungs in the sham group (Fig 6E). In summary, RLIPC-induced protection against lung I/R injury was highly STAT3-dependent.

## Discussion

Pulmonary I/R injury is an established clinical problem that adversely affects outcomes and increases mortality following lung transplantation. There is therefore an urgent need for therapeutic strategies that mitigate pulmonary I/R injury. Unlike unexpected events that also involve ischemia and require subsequent reperfusion, e.g., pulmonary embolism or acute myocardial infarction, lung transplantation is a planned, invasive, I/R event. In other words, both the ischemic (especially in the case of a living donor) and the reperfusion phases of a lung transplantation are expected and their timing is to some extent controlled. This distinction has important ramifications when it comes to therapy, as it means that planned, invasive therapeutic interventions may be possible prior to lung transplantation. Thus, therapies that might be ineffective if applied post-ischemia, or which are too invasive in other scenarios, might be applicable in the case of lung transplantation.

To explore one possible avenue, we examined whether RLIPC, an approach we previously found to be effective in attenuating cardiac I/R-induced infarct size and arrhythmia severity [13, 16], might be similarly effective in protecting the lungs. Using a range of indicators, including structural damage scoring, edema quantification, apoptosis markers, blood gases and inflammatory markers, we demonstrate that RLIPC is indeed an effective protective strategy in the rat model of pulmonary I/R injury used herein.

Pulmonary I/R injury, as with many other injurious, non-infectious events including physical trauma or chemically induced damage, involves a major “sterile inflammation” component in which inflammation is induced by activation of the innate immune system[8]. TNF-α and IL-6 are important mediators of innate immune responses to injury, are produced in response to pulmonary I/R, and are associated with I/R-induced damage to the lung [8, 19, 20]. Previous work showed that hepatic preconditioning reduced TNF-α production and hepatic damage caused by subsequent hepatic I/R injury, and that this in turn attenuated lung injury consequent to hepatic I/R, presumably by attenuating systemic TNF-α release from the liver [26]. We now show that a similar reduction in the innate immune response, and pulmonary production of TNF-α and IL-6, is observed when the hepatic preconditioning is remote to the site of I/R injury, which in the present study was in the lung. One interesting possibility, since the lung is a site of platelet biogenesis and a reservoir for hematopoietic progenitors [27], is that RLIPC leads to a rapid mobilization of platelets from resident megakaryocytes in the lung prior to the subsequent severe ischemic insult, resulting in a reduction of the inflammatory markers/cells in the lung. While RLIPC itself is unlikely to represent a viable procedure in donors or recipients involved in lung transplantation, our studies uncovered STAT3 as an essential factor in RLIPC-induced pulmonary protection, and this does present possible future therapeutic avenues.

There has been much interest in the roles of the reperfusion injury salvage kinase (RISK) and survivor activating factor enhancement (SAFE) signaling pathways in protection against I/R injury, and particularly in myocardial I/R injury. The RISK pathway, which comprises protein kinase B (Akt) and extracellular signal-regulated kinases (ERK1/2), is reportedly crucial for the cardioprotective effects of both local and remote ischemic preconditioning [28]. Ischemic conditioning stimuli lead to ERK1/2 or Akt phosphorylation and thus activation, and reduced myocardial infarct size [29]. Furthermore, phosphorylation of GSK-3β(Ser9), a downstream target of Akt and ERK1/2, inhibitsGSK-3β and enhances myocardial survival against I/R [16]. This protective phenomenon is mediated through inhibition of mitochondrial permeability transition pore (mPTP) opening and myocyte apoptosis [30]. The SAFE pathway, which includes the transcription factor STAT3, is independent of the RISK pathway, but its activation is also cardioprotective in the context of ischemic conditioning [31]. Accordingly, inhibiting the SAFE pathway can abolish protective effects of ischemic pre- or post-conditioning [32].

Here, we did not find evidence of a role for RISK pathway activation in RLIPC-induced pulmonary protection against I/R injury. In contrast, we observed increased phospho-STAT3 levels in the lungs of RLIPC rats compared to controls (or shams). There is STAT3 activation above baseline in the control group, but this is further increased in the RLIPC group. What makes the relatively higher level of STAT3 activation above baseline we observe in the RLIPC group versus the control group confer protection in the former is not known; it may reach a tipping point in the RLIPC group for activation of some other pathway. Nevertheless, because pharmacological inhibition of STAT3 phosphorylation negated the pulmonary protection afforded by RLIPC, we can conclude that STAT3 activation, likely as part of the SAFE pathway but independent of the RISK pathway, was necessary (if not sufficient) for RLIPC-induced protection against pulmonary I/R injury. This is in line with a recent report demonstrating a causal role for STAT3, but not Akt or ERK1/2, activation in the cardioprotective effects of ischemic preconditioning in pigs [25]. However, it contrasts with the recent report that STAT3 inhibition is required for the protective effect of SO_2_ in an acute lung injury model induced by limb ischemia/reperfusion in rats [33]. This could indicate that the relative benefits of STAT3 induction are highly context-dependent, and depend on the cause of lung injury. In addition, our current findings for lung preconditioning by RLIPC contrast with our recent findings for myocardial pre- and post-conditioning using RLIPC. We found activation of STAT5, in control rats and to a higher degree in RLIPC rats. STAT5 activation is thought to be important in cardioprotection, and perhaps specifically in cardioprotection afforded by RIPC [23, 24]. Previously, STAT5 activation was found only in patients who underwent RIPC, and not in control patients, in contrast to our rat data [24]. However, as we are examining a different species and a different tissue, we cannot comment on reasons underlying this discrepancy, beyond these obvious differences.

Phosphorylation of GSK-3β serine 9 inhibits GSK-3β activity [34]. We found that either pre- or post-conditioning, or a combination of both, using RLIPC was both highly cardioprotective in the context of myocardial I/R injury, and required GSK-3β serine 9 phosphorylation, thus implicating RISK pathway induction [16]. Similarly, we found that in both diabetic rats and non-diabetic rats, RLIPC reduced the incidence of lethal arrhythmias, induced RISK pathway activation (stimulation of ERK1/2, Akt and GSK-3β phosphorylation) and was GSK-3β serine 9 phosphorylation-dependent [12, 13]. In the present study, we observed no change in lung ERK1/2, Akt, or GSK-3β serine 9 phosphorylation following RLIPC.

In a lung transplant model that mimicked in mice the I/R-induced acute graft injury found in human lung transplant recipients, STAT3 was required for the beneficial effect of the transcriptional co-regulator B cell leukemia/lymphoma 3 (Bcl3) in preventing acute inflammatory lung injury [35]. Bcl3 was required for limiting emergency granulopoiesis, but only in the lung I/R injury model (in which Bcl3 limited acute graft damage by attenuating granulopoiesis), and not under baseline conditions. These data are consistent with our findings of a protective role for STAT3 against lung injury when the injury is caused by I/R in the lung itself and not a remote I/R as was used in the SO_2_ study described above [33]. They also point to a potential direction for further work, to investigate whether Bcl3 plays a role in the RLIPC preconditioning we observed in the present study. In addition, additional studies can be directed toward understanding if pharmacological STAT3 activation could be optimized and employed to provide analogous pulmonary protective effects to RLIPC, circumventing the invasive procedure but retaining the beneficial molecular component.

We acknowledge potential limitations of this study. First, we only tested ERK, AKT and GSK-3β Ser9 within the RISK pathway and STAT3 and 5 in the JAT/STAT pathway; other signaling molecules may also participate in this pulmonary protective cascade beyond the activation of STAT3. Therefore, future studies could address detailed pathway involvement in liver ischemic preconditioning-induced anti-pulmonary IR injury, and if there is any crosstalk in this context within these signaling pathways. In addition, we did not yet test the effects of inhibition of STAT5 or other possible protective components, focusing here instead on STAT3. Second, immune cells may play a role in this protective phenomenon, whose recruitment may be modulated by chemotactic effects post liver stimuli. Meanwhile, other mechanisms may also be included as cytokines secretion, endogenous neural or humoral agent(s) releasing and further travelling from remote organ to the lungs, exerting anti-apoptosis and anti-inflammatory effects in the lungs. Third, four cycles of 5 min ischemia/5 min reperfusion liver preconditioning ischemic intervention protocol were adopted in our study; it remains unknown whether other liver preconditioning stimuli (sustained preconditioning), or liver ischemic postconditioning strategies can produce lung protection.

## Supporting information

S1 Fig Raw data. Raw data including entire western blots and micrographs are shown, with subheadings indicating their corresponding figure numbers and panels in the main figures.
(PDF)
