# Supplementary material for: Remote ischemic preconditioning STAT3-dependently ameliorates pulmonary ischemia/reperfusion injury
Source: PLoS One. 2018 May 16;13(5):e0196186. doi: 10.1371/journal.pone.0196186 (PMC5955491; doi:10.1371/journal.pone.0196186)
Supplement: S1 Fig — Raw data including entire western blots and micrographs are shown, with subheadings indicating their corresponding figure numbers and panels in the main figures. (PDF) [file pone.0196186.s001.pdf]

Western blot data:

Figure 5A

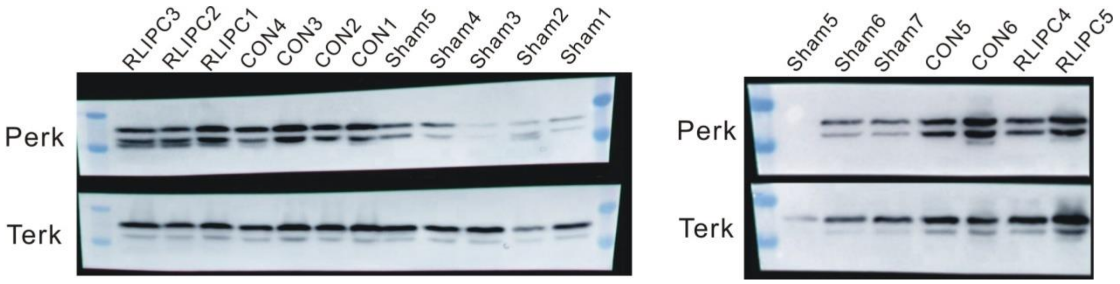

Figure 5B

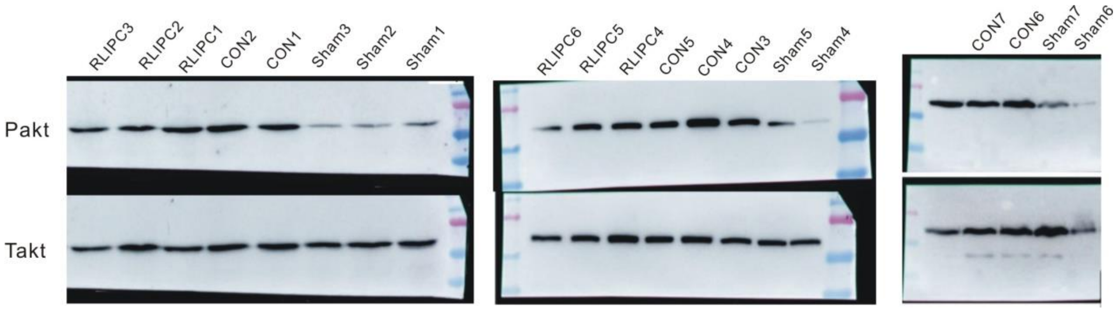

Figure 5C

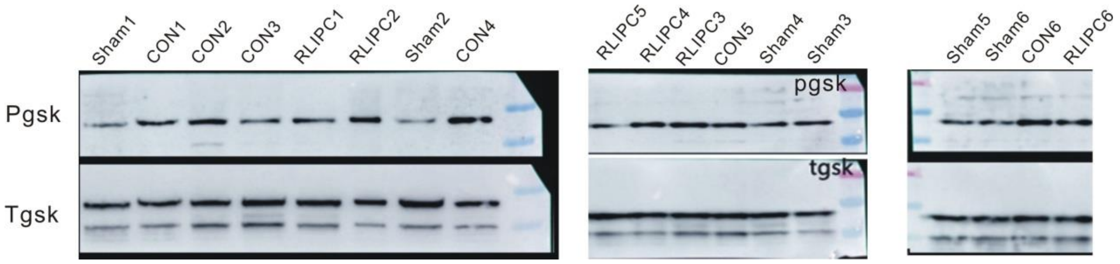

Figure 5D

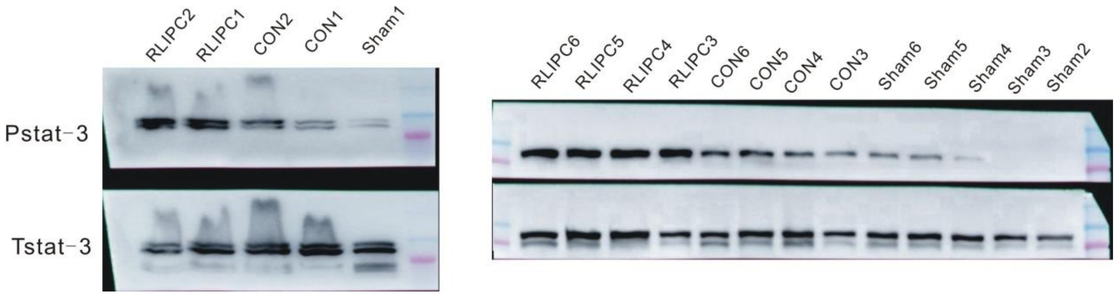

Figure 5E

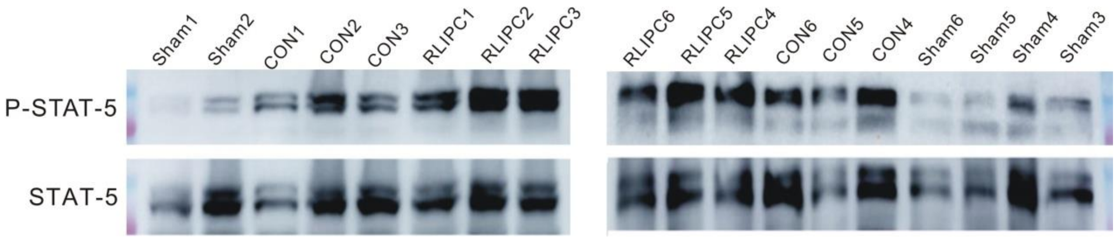

Figure 4B

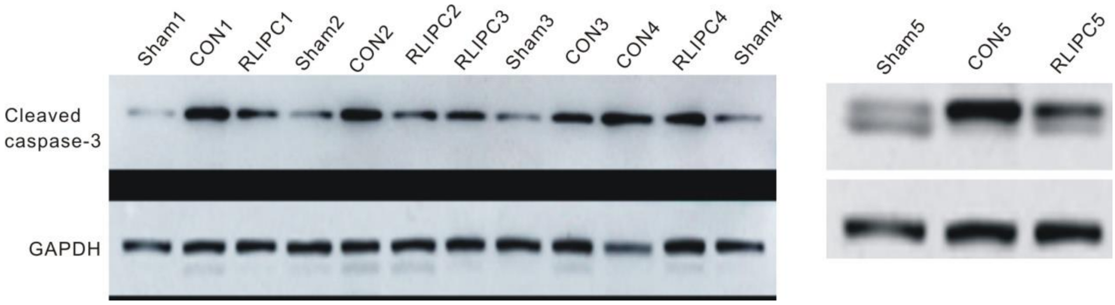

Figure 6E

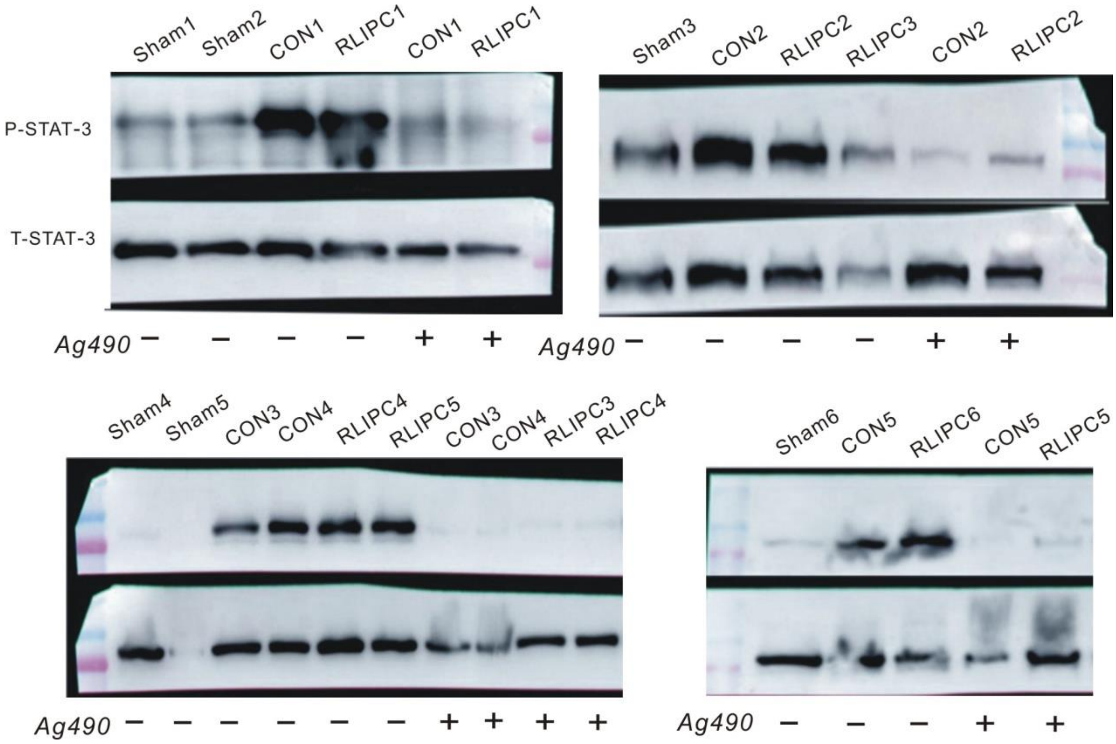

Histology data:

Figure 2A

10x sham

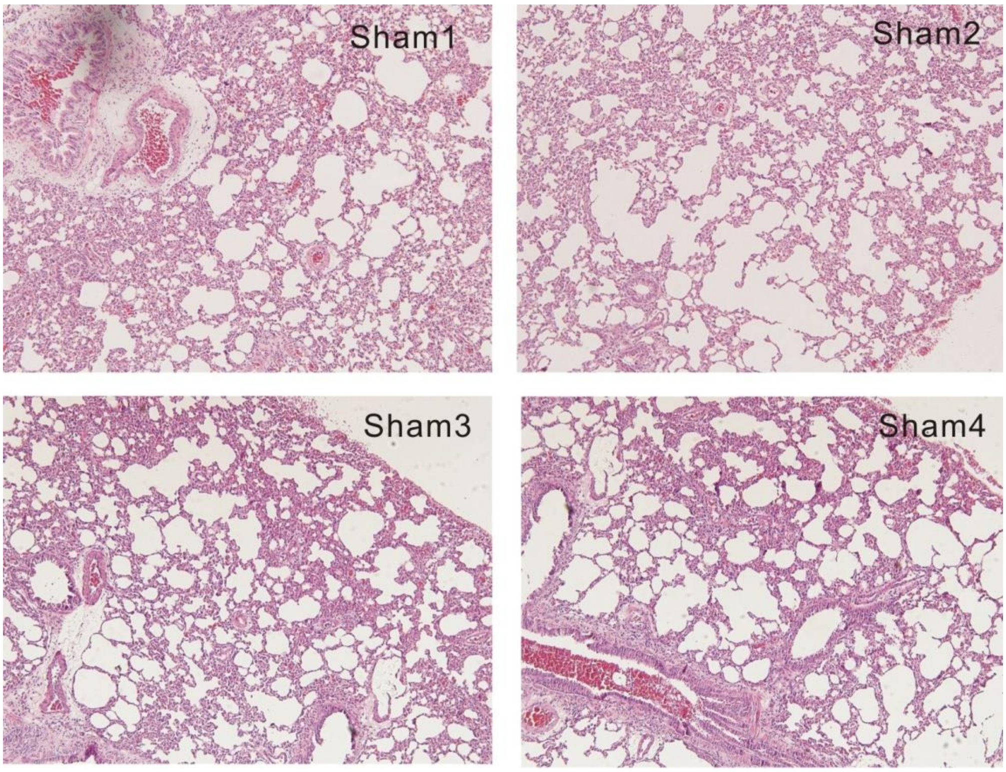

40x sham

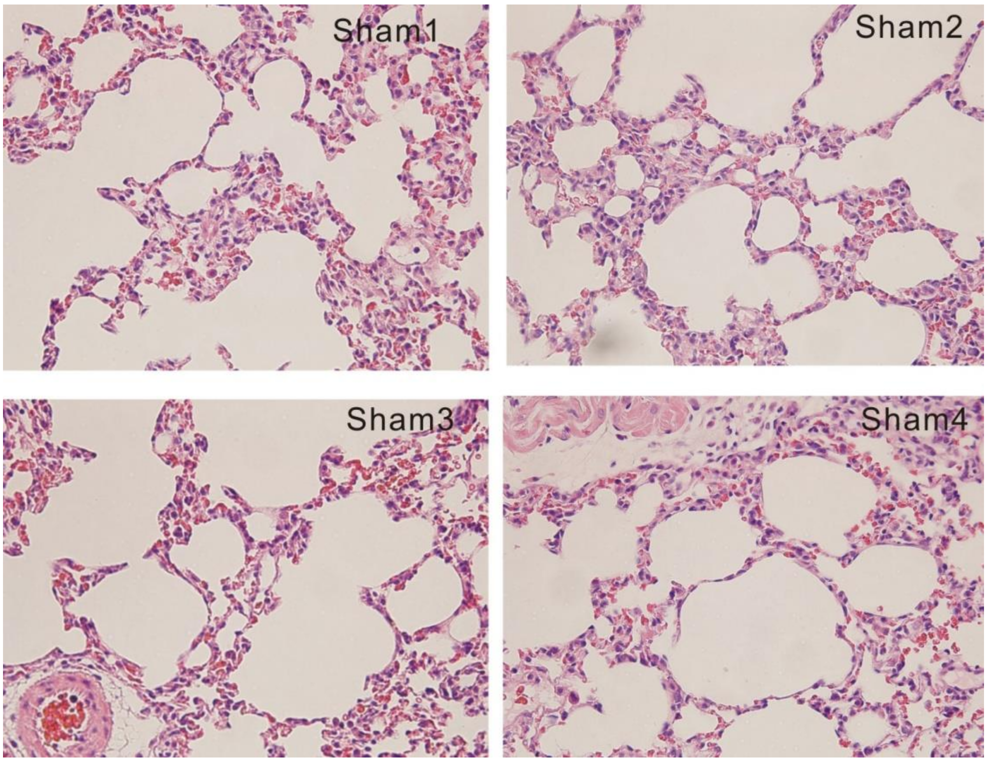

10x CON

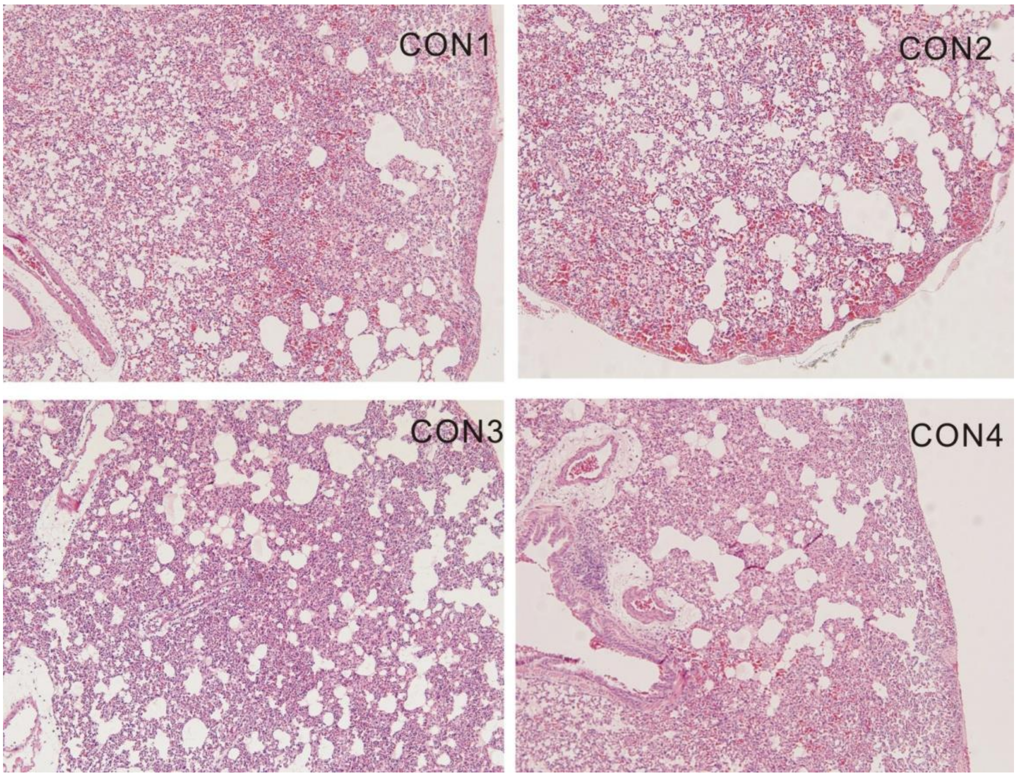

40x CON

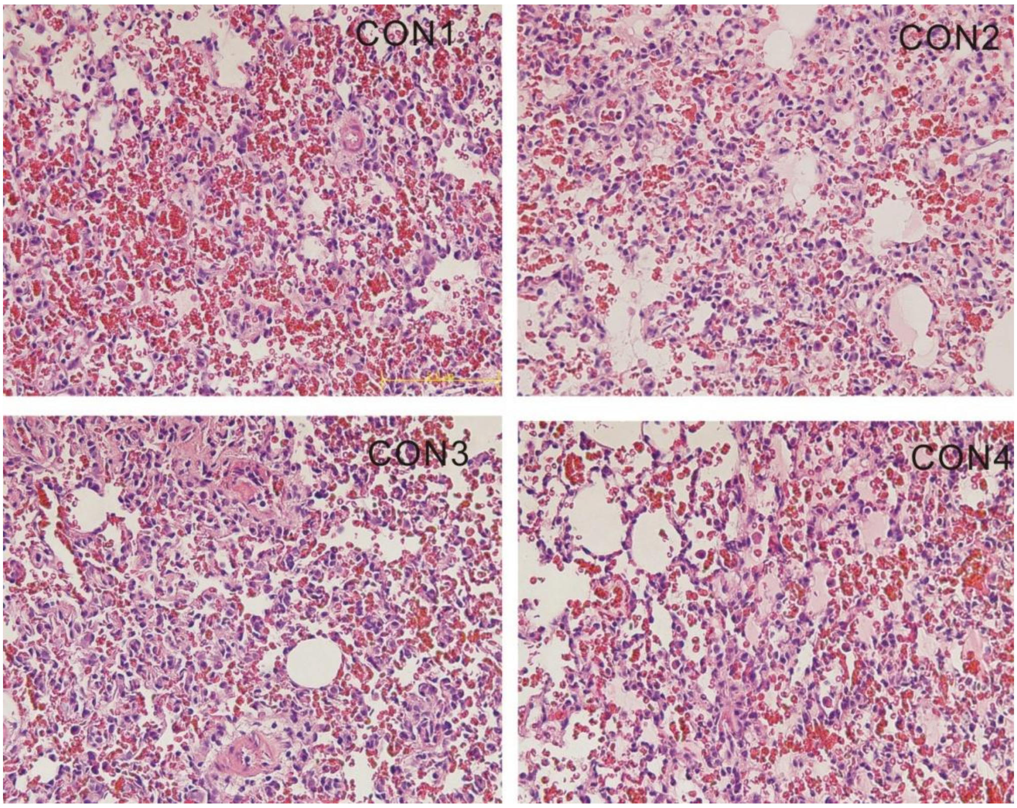

10x RLIPC

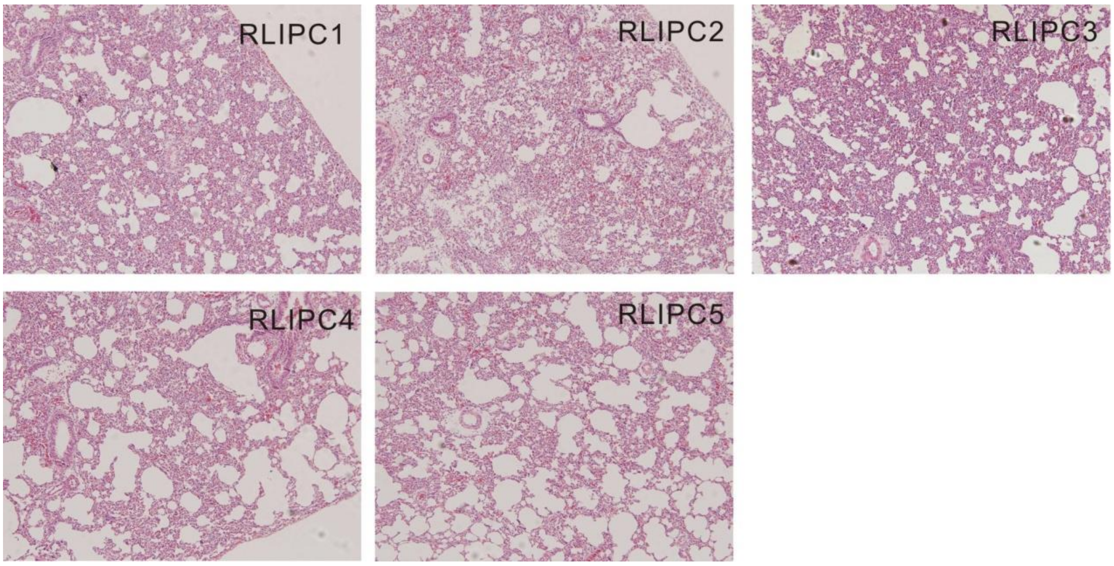

40x RLIPC

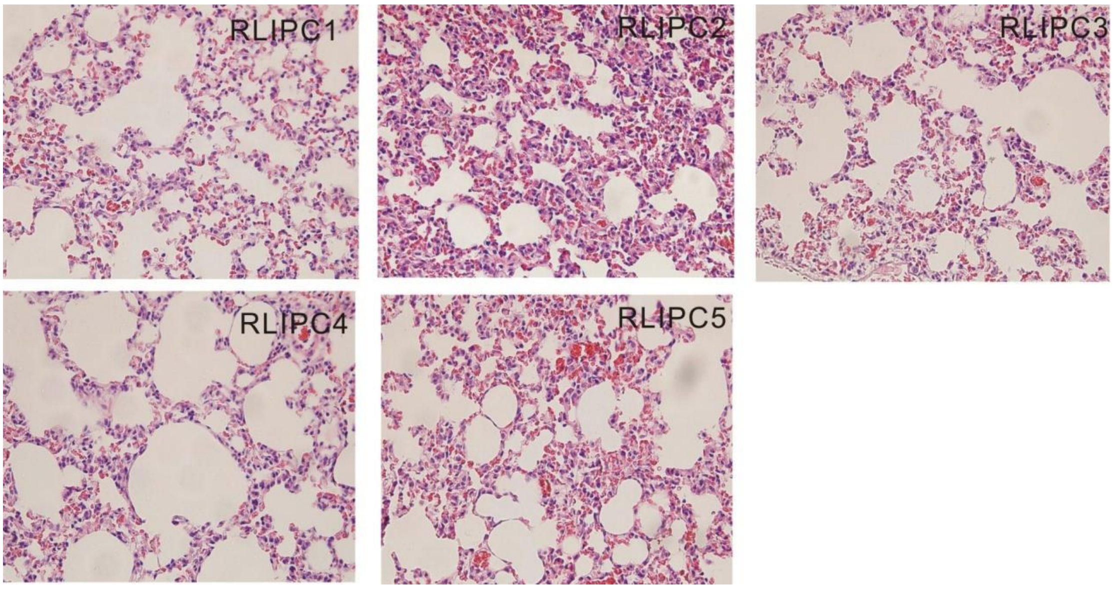

Figure 6D

40x AG490 CON

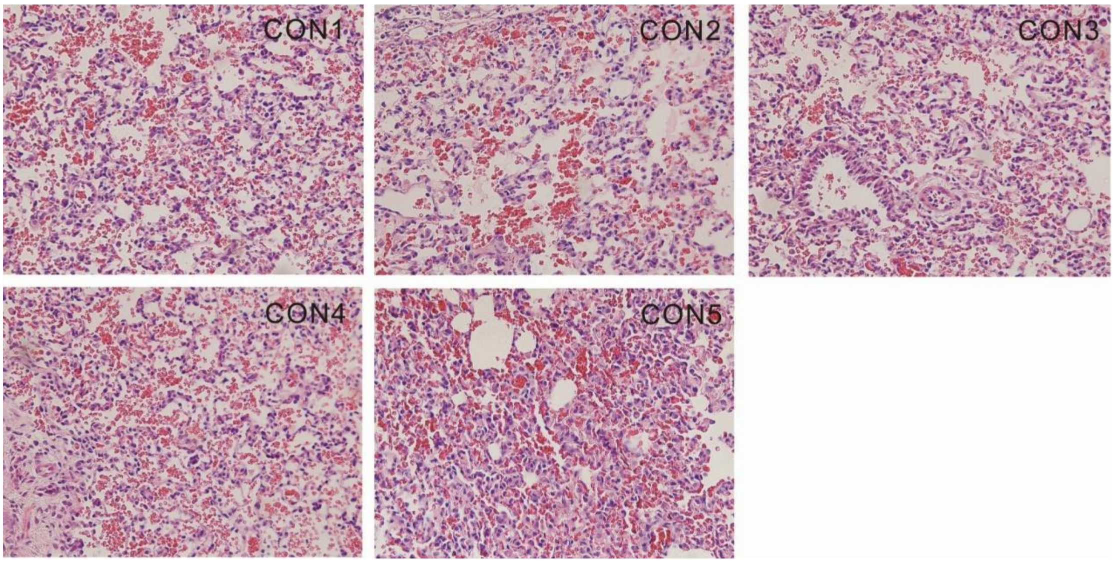

40x AG490 RLIPC

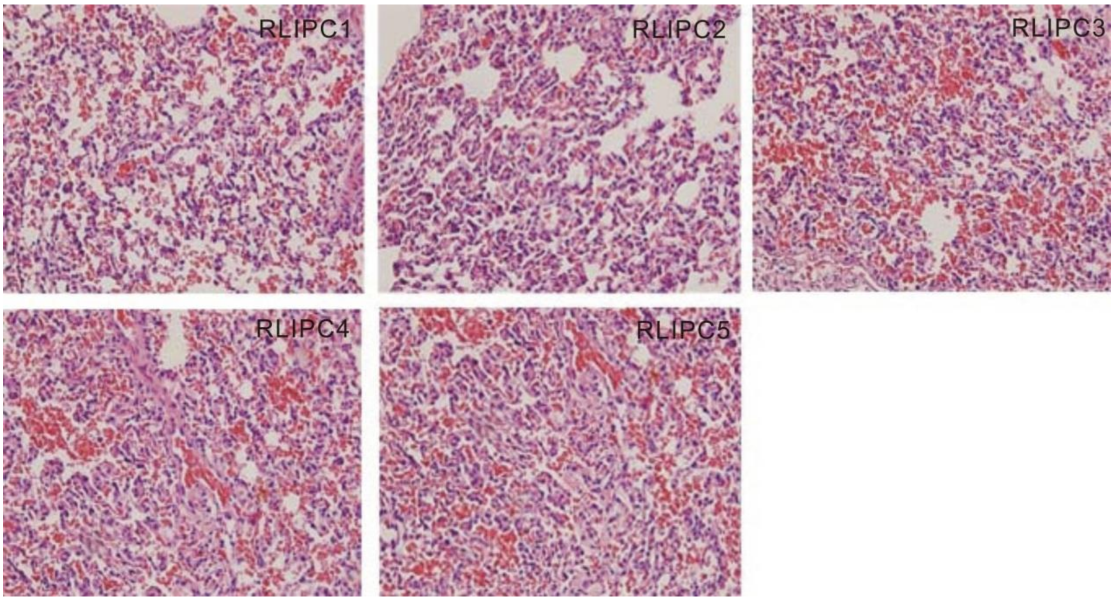

Figure 4A

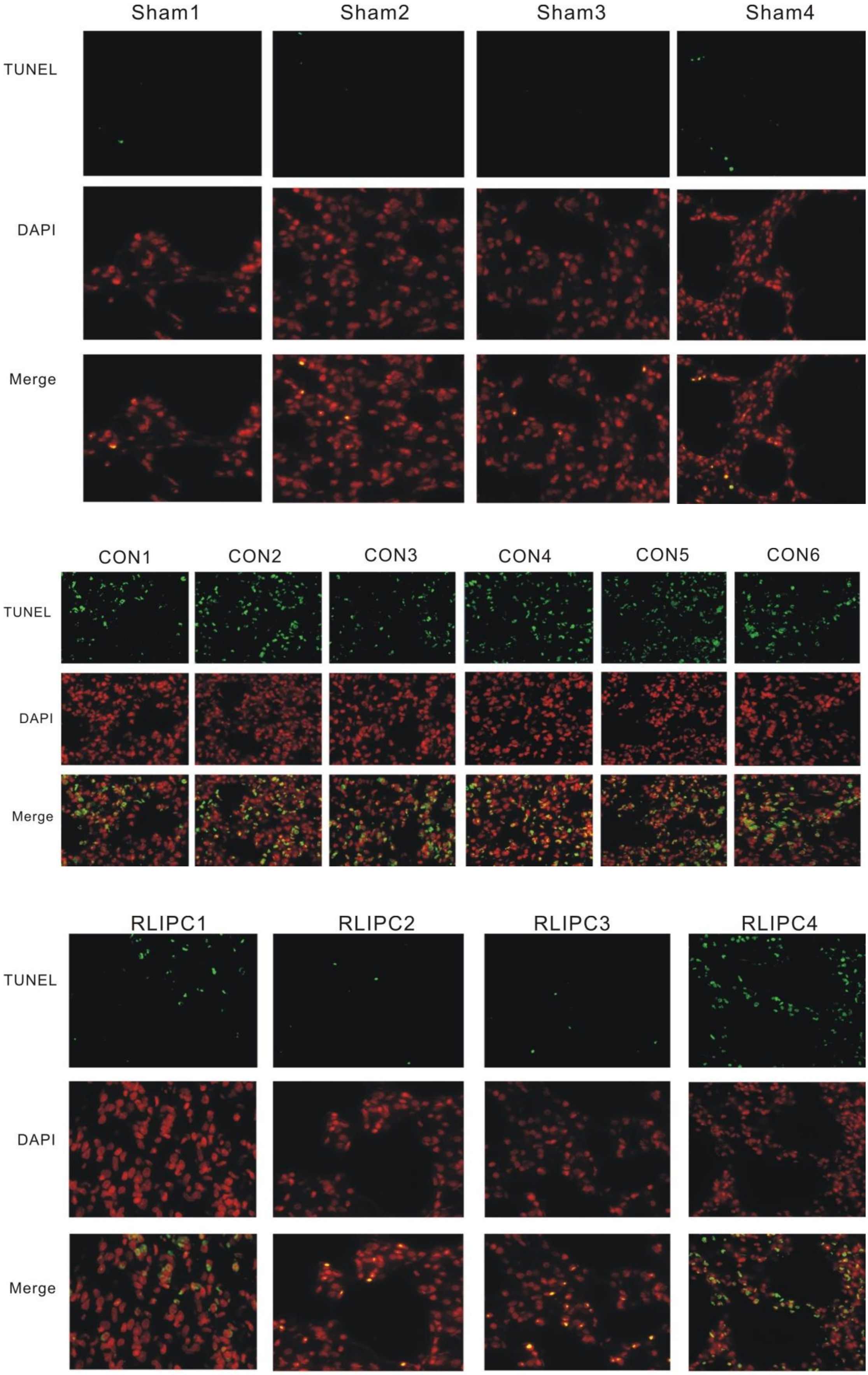
Sham1
Sham2
Sham3
Sham4
TUNEL
DAPI
Merge
CON1
CON2
CON3
CON4
CON5
CON6
TUNEL
DAPI
Merge
RLIPC1
RLIPC2
RLIPC3
RLIPC4
TUNEL
DAPI
Merge
